# Three dimensional accuracy and stability of segmented Le Fort I osteotomy – a systematic review

**DOI:** 10.1186/s13005-026-00625-x

**Published:** 2026-06-26

**Authors:** Oliver da Costa Senior, Marie Coppens, Reinhilde Jacobs, Eman Shaheen, Constantinus Politis

**Affiliations:** 1 Department of Imaging and Pathology, Faculty of Medicine, OMFS IMPATH Research Group, KU Leuven, Louvain, Belgium; 2 Department of Oral and Maxillofacial Surgery, University Hospitals Leuven, Louvain, Belgium; 3 Department of Maxillofacial Surgery, ZMACK, AZ MONICA Antwerpen, Antwerp, Belgium; 4 Department of Cranio-Maxillofacial Surgery, Antwerp University Hospital, Edegem, Belgium; 5 Faculty of Medicine & Health Sciences, University of Antwerp, Campus Drie Eiken, Antwerp, Belgium; 6 All for Research Vzw, Antwerp, Belgium; 7 Department of Dental Medicine, Karolinska Institutet, Stockholm, Sweden

**Keywords:** Orthognathic surgery, Computer-aided design, Imaging, three-dimensional, Osteotomy, Le Fort, Segmented Le Fort I

## Abstract

**Background:**

Segmental Le Fort I osteotomy has traditionally been regarded as less predictable and stable, a perception largely based on two-dimensional evaluation methods. Despite existing systematic reviews on clinical outcomes, a dedicated synthesis of three-dimensional assessments of surgical accuracy and postoperative stability is lacking.

**Purpose:**

This systematic review evaluated three-dimensional (3D) assessment methods and outcome of planning accuracy and postoperative stability in segmented Le Fort I osteotomies. The aim was to determine the reliability of 3D methods in capturing surgical accuracy and long-term skeletal stability.

**Study selection:**

Medline, Embase, Web of Science, and Cochrane were searched up to October 25, 2024. Eligible studies included randomized controlled trials, prospective or retrospective studies, or case series with > 5 patients reporting 3D evaluation of planning accuracy or stability. Exclusion criteria were systematic reviews, meta-analyses, single case reports, ex-vivo or animal studies, and studies involving syndromic patients, facial trauma, or prior maxillofacial surgery. Risk of bias and quality of evidence were assessed using the GRADE approach. Twenty-two studies met inclusion criteria: three randomized and nineteen non-randomized controlled trials. Fourteen assessed planning accuracy, five evaluated stability, and three examined both.

**Results:**

Twenty-two studies met the inclusion criteria and were included in the qualitative synthesis. Considerable methodological heterogeneity limited direct comparison and precluded meta-analysis. Overall, segmented Le Fort I osteotomy demonstrated clinically acceptable accuracy; however, certain movements—particularly transverse widening, advancement, and pitch—were more prone to underachievement, with discrepancies ranging from 0.77 to 1.41 mm, 0.55 to 2.69 mm, and 0.12° to 5.22°, respectively. During follow-up, transverse relapse was more pronounced at the dental level (0.39–1.41 mm) compared to skeletal changes (0.05–0.86 mm).

**Conclusion:**

Segmented Le Fort I osteotomy shows reliable outcomes in 3D evaluations, but some maxillary movements remain difficult to achieve and maintain such as advancement and pitch. Standardized, voxel-based 3D assessment protocols are recommended to improve comparability and strengthen the evidence base. (PROSPERO registration: CRD42018111854).

## Introduction

Segmental Le Fort I osteotomy enables correction of transverse maxillary discrepancies in addition to vertical, sagittal, and rotational adjustments. Despite its versatility, concerns remain regarding postoperative relapse, particularly in cases of large transverse expansion [1, 2]. Historically, this skepticism has been based on two-dimensional (2D) postero-anterior radiographs and dental cast evaluations [3–7], both of which provide limited insight into complex skeletal changes.

A previous systematic review by Haas et al. [7] evaluated surgical complications and postoperative stability (without reporting surgical accuracy) of segmented Le Fort I osteotomy up to 2016. However, this review predominantly included studies relying on 2D cephalometry and dental cast analysis, with only a single study by Yao et al. [8] applying three-dimensional (3D) cone-beam computed tomography (CBCT) imaging for stability assessment. In addition, studies employing non-rigid fixation techniques were included, further contributing to heterogeneity in reported outcomes. As a result, the available evidence summarized in this review does not fully capture the three-dimensional complexity of segmented maxillary movements.

Orthognathic outcomes hinge on three critical steps [9]: (1) accurate diagnosis and preoperative planning [10]; (2) precise intraoperative transfer of the plan, achieved through splints, patient-specific plates, or real-time navigation; and (3) postoperative stability, especially following maxillary widening [11, 12]. To fully understand outcomes, both planning accuracy and long-term stability must be assessed, as these reveal potential causes of relapse and inform refinements in treatment planning.

The advent of three-dimensional (3D) imaging has transformed this process, enhancing diagnostic precision, surgical planning, and plan-to-surgery transfer. Over the past decade, 3D evaluation of surgical accuracy and postoperative stability has been increasingly adopted, enabling a more comprehensive and precise assessment of orthognathic outcomes. Multiple studies have shown 3D planning to be superior—or at least equivalent—to 2D methods for one-piece Le Fort I osteotomy [13–16]. Moreover, 3D imaging allows more detailed evaluation of relapse in complex maxillary movements, such as transverse expansion, translation or rotational yaw movements [10, 17, 18].

Various 3D protocols have been proposed, including registration-free and registration-based methods, with the latter subdivided into landmark-, surface-, and voxel-based approaches [19–22]. A universal 3D assessment protocol has been advocated based on voxel-based registration, (semi-)automated outcome assessment and reporting of inter- and intra-observer reliability [23, 24]. These methodological advancements allow a more robust and reproducible evaluation of surgical outcomes compared to traditional 2D techniques.

Segmented Le Fort I osteotomy (sLFI) introduces additional complexity, as each maxillary segment is repositioned in multiple dimensions. This makes robust 3D assessment even more critical for evaluating intraoperative accuracy, postoperative stability, and ultimately, for determining appropriate indications and providing reliable patient counseling.

Despite these developments, no systematic review to date has specifically focused on the three-dimensional assessment of surgical planning accuracy and postoperative stability in segmented Le Fort I osteotomy. By exclusively evaluating studies using 3D methodologies, the present review aims to provide a more detailed and clinically relevant understanding of surgical outcomes, thereby addressing a gap in the current literature.

The primary objective of this systematic review was to evaluate the three-dimensional (3D) surgical outcomes of sLFI. The secondary objective was to critically appraise the 3D methods employed in the included studies for assessing the accuracy of virtual surgical planning and long-term stability.

## Materials and methods

### Eligibility criteria

Studies were eligible for inclusion if they were randomized controlled trials, prospective or retrospective clinical studies, or case series involving more than five sLFI patients, and if they reported on planning accuracy or stability. Only studies that used 3D tools to assess outcomes were considered. Full-text articles had to be available in English, French, German, or Dutch.

Exclusion criteria included systematic reviews or meta-analyses, single case reports, ex-vivo or animal studies, and studies involving syndromic patients, previous facial trauma, or prior maxillofacial surgery.

### Information sources

The electronic databases MEDLINE via PubMed, Embase, Web of Science, and the Cochrane Central Register of Controlled Trials were searched up to October 25, 2024.

### Search strategy

The search strategy was developed in collaboration with the Désiré Collen Learning Center Library, Catholic University of Leuven, following the Patient, Intervention, Comparison, and Outcome (PICO) framework. The population and intervention were defined as patients undergoing sLFI. The comparison included different 3D assessment methods, and the outcome was defined as maxillary planning accuracy and stability. The specific search terms used for each database are provided in Table 1.

**Table 1:** Medline, Embase, Web of Science and Cochrane search terms

MEDLINE via Pubmed	
"orthognathic surgery"[MeSH Terms] OR orthognathic surg*[tiab] OR jaw surg*[tiab] OR "orthognathic surgical procedures"[MeSH Terms] OR "osteotomy, le fort"[MeSH Terms] OR "le fort I"[tiab] OR “lefort I”[tiab] OR "maxillary osteotomy"[MeSH Terms] OR maxillary osteotom*[tiab] OR "open bite"[MeSH Terms] OR "segmented osteotomy"[tiab] OR segmented osteotom*[tiab] OR "multipiece le fort I"[tiab] OR "2 piece le fort"[tiab] OR "3 piece le fort"[tiab] OR "three piece le fort"[tiab] OR "maxillary segmentation"[tiab] OR maxillary segment*[tiab] OR segment* le fort [tiab]	
AND "Cone-Beam Computed Tomography"[Mesh] OR "CBCT"[tiab] OR Cone-Beam Computed Tomograph*[tiab] OR "Image Processing, Computer-Assisted"[Mesh] OR "Imaging, Three-Dimensional"[Mesh] OR "3D"[tiab] OR "3-D"[tiab] OR " 3-Dimensional"[tiab] OR "User-Computer Interface"[Mesh] OR "three D"[tiab] OR "Computer-Aided Design"[Mesh] OR "CAD/CAM"[tiab] OR "Printing, Three-Dimensional"[Mesh] OR "Stereolithography"[Mesh] OR "Radiographic Image Interpretation, Computer-Assisted"[Mesh] OR "Surgery, Computer-Assisted"[Mesh] OR computer assisted surg*[tiab] OR computer aided surg*[tiab] OR "Computer Simulation"[Mesh] OR "Computer-Aided Surgical Simulation"[tiab] OR "Surgical Simulation"[tiab] OR "Three-dimensional analysis"[tiab] OR "VSP"[tiab] OR "virtual surgical planning"[tiab] OR "virtual planning"[tiab] OR "intraoperative navigation"[tiab] OR surgical plan*[tiab] OR "surgical splint"[tiab] OR "intermediate splint"[tiab]	
AND "Recurrence"[Mesh] OR “Recrudescence”[tiab] OR "Treatment Outcome"[Mesh] OR “effectiveness”[tiab] OR “efficacy”[tiab] OR "Treatment Failure"[Mesh] OR "Outcome Assessment (Health Care)"[Mesh] OR "Patient Outcome Assessment"[Mesh] OR "patient outcome"[tiab] OR "Dimensional Measurement Accuracy"[Mesh] OR "Reproducibility of Results"[Mesh] OR “Predictability”[tiab] OR "Vertical Dimension"[Mesh] OR "Data Accuracy"[Mesh] OR "accuracy"[tiab] OR "accurate "[tiab] OR "precision"[tiab] OR "Long Term Adverse Effects"[Mesh] OR long term adverse effect*[tiab] OR "Bone Remodeling"[Mesh] OR bone remodel*[tiab] OR “Patient Satisfaction”[Mesh] OR “Patient Preference”[Mesh] OR “Relapse”[tiab] OR "stability"[tiab] OR "skeletal stability"[tiab] OR "dental stability"[tiab] OR "reliability"[tiab] OR "surgical outcome"[tiab]	

**Embase**	
'orthognathic surgery'/exp OR ‘orthognathic surg*’:ab,ti OR ‘jaw surg*’:ab,ti OR 'maxillofacial surgery'/exp OR 'maxilla osteotomy'/exp OR 'Le Fort osteotomy'/exp OR ‘Le Fort I osteotomy’/exp OR ‘le fort I’:ab,ti OR ‘lefort I’:ab,ti OR ‘maxillary osteotom*’:ab,ti OR ‘open bite’:ab,ti OR ‘segmented osteotomy’:ab,ti OR ‘segmented osteotom*’:ab,ti OR ‘multipiece le fort I’:ab,ti OR ‘2 piece le fort’:ab,ti OR ‘3 piece le fort’:ab,ti OR ‘three piece le fort’:ab,ti OR ‘maxillary segmentation’:ab,ti OR ‘maxillary segment*’:ab,ti OR ‘segment* le fort’:ab,ti	
AND 'cone beam computed tomography'/exp OR 'three dimensional imaging'/exp OR 'computer interface'/exp OR 'computer aided design'/exp OR 'three dimensional printing'/exp OR 'stereolithography'/exp OR 'computer assisted surgery'/exp OR 'computer simulation'/exp OR ‘CBCT’:ab,ti OR ‘Cone-Beam Computed Tomograph*’:ab,ti OR ‘3D’:ab,ti OR ‘3-Dimensional’:ab,ti OR ‘three D’:ab,ti OR ‘3-D’:ab,ti OR ‘CAD/CAM’:ab,ti OR ‘computer assisted surg*’:ab,ti OR ‘computer aided surg*’:ab,ti OR ‘Computer-Aided Surgical Simulation’:ab,ti OR ‘Surgical Simulation’:ab,ti OR ‘Three-dimensional analysis’:ab,ti OR ‘VSP’:ab,ti OR ‘virtual surgical planning’:ab,ti OR ‘virtual planning’:ab,ti OR ‘intraoperative navigation’:ab,ti OR ‘surgical plan*’:ab,ti OR ‘surgical splint’:ab,ti OR ‘intermediate splint’:ab,ti	
AND 'relapse'/exp OR 'recurrent disease'/exp OR 'treatment outcome'/exp OR 'clinical outcome'/exp OR 'patient-reported outcome'/exp OR 'treatment failure'/exp OR 'outcome assessment'/exp OR ‘measurement accuracy'/exp OR 'reproducibility'/exp OR 'vertical dimension of occlusion'/exp OR 'patient satisfaction'/exp OR 'patient preference'/expOR ‘Recrudescence’:ab,ti OR ‘effectiveness’:ab,ti OR ‘efficacy’:ab,ti OR ‘patient outcome’:ab,ti OR ‘Predictability’:ab,ti OR ‘accuracy’:ab,ti OR ‘accurate ‘:ab,ti OR ‘precision’:ab,ti OR ‘long term adverse effect*’:ab,ti OR 'bone remodeling'/exp OR ‘bone remodel*’:ab,ti OR ‘Relapse’:ab,ti OR ‘stability’:ab,ti OR ‘skeletal stability’:ab,ti OR ‘dental stability’:ab,ti OR ‘reliability’:ab,ti OR ‘surgical outcome’:ab,ti	

**Web of science**	
TS = (orthognathic surgery OR orthognathic surg* OR jaw surg* OR orthognathic surgical procedures OR osteotomy, le fort OR le fort I OR lefort I OR maxillary osteotomy OR maxillary osteotom* OR open bite OR segmented osteotomy OR segmented osteotom* OR multipiece le fort I OR 2 piece le fort OR 3 piece le fort OR three piece le fort OR maxillary segmentation OR maxillary segment* OR segment* le fort)	
AND TS = (Cone-Beam Computed Tomography OR CBCT OR Cone-Beam Computed Tomograph* OR Image Processing, Computer-Assisted OR Imaging, Three-Dimensional OR 3D OR “3-D” OR “three D” OR 3-Dimensional OR User-Computer Interface OR Computer-Aided Design OR CAD/CAM OR Printing, Three-Dimensional OR Stereolithography OR Radiographic Image Interpretation, Computer-Assisted OR Surgery, Computer-Assisted OR computer assisted surg* OR computer aided surg* OR Computer Simulation OR Computer-Aided Surgical Simulation OR Surgical Simulation OR Three-dimensional analysis OR VSP OR virtual surgical planning OR virtual planning OR intraoperative navigation OR surgical plan* OR surgical splint OR intermediate splint)	
AND TS = (Recurrence OR Recrudescence OR Treatment Outcome OR effectiveness OR efficacy OR Treatment Failure OR Outcome Assessment (Health Care) OR Patient Outcome Assessment OR patient outcome OR Dimensional Measurement Accuracy OR Reproducibility of Results OR Predictability OR Vertical Dimension OR Data Accuracy OR accuracy OR accurate OR precision OR Long Term Adverse Effects OR long term adverse effect* OR Bone Remodeling OR bone remodel* OR Patient Satisfaction OR Patient Preference OR Relapse OR stability OR skeletal stability OR dental stability OR reliability OR surgical outcome)	

**Cochrane**	
"orthognathic surgery"[MeSH Terms] OR orthognathic surg*[tiab] OR jaw surg*[tiab] OR "orthognathic surgical procedures"[MeSH Terms] OR "osteotomy, le fort"[MeSH Terms] OR "le fort I"[tiab] OR “lefort I”[tiab] OR "maxillary osteotomy"[MeSH Terms] OR maxillary osteotom*[tiab] OR "open bite"[MeSH Terms] OR "segmented osteotomy"[tiab] OR segmented osteotom*[tiab] OR "2 piece le fort"[tiab] OR "3 piece le fort"[tiab] OR "three piece le fort"[tiab] OR maxillary segment*[tiab] OR segment* le fort [tiab]	
AND "Cone-Beam Computed Tomography"[Mesh] OR "CBCT"[tiab] OR Cone-Beam Computed Tomograph*[tiab] OR "Image Processing, Computer-Assisted"[Mesh] OR "Imaging, Three-Dimensional"[Mesh] OR "3D"[tiab] OR "3-D"[tiab] OR "three D"[tiab] OR " 3-Dimensional"[tiab] OR "User-Computer Interface"[Mesh] OR "Computer-Aided Design"[Mesh] OR "CAD/CAM"[tiab] OR "Printing, Three-Dimensional"[Mesh] OR "Radiographic Image Interpretation, Computer-Assisted"[Mesh] OR "Surgery, Computer-Assisted"[Mesh] OR computer assisted surg*[tiab] OR computer aided surg*[tiab] OR "Computer Simulation"[Mesh] OR "Computer-Aided Surgical Simulation"[tiab] OR "Surgical Simulation"[tiab] OR "Three-dimensional analysis"[tiab] OR "VSP"[tiab] OR "virtual surgical planning"[tiab] OR "virtual planning"[tiab] OR "intraoperative navigation"[tiab] OR surgical plan*[tiab] OR "surgical splint"[tiab] OR "intermediate splint"[tiab]	
AND "Recurrence"[Mesh] OR “Recrudescence”[tiab] OR "Treatment Outcome"[Mesh] OR “effectiveness”[tiab] OR “efficacy”[tiab] OR "Treatment Failure"[Mesh] OR "Outcome Assessment (Health Care)"[Mesh] OR "Patient Outcome Assessment"[Mesh] OR "patient outcome"[tiab] OR "Dimensional Measurement Accuracy"[Mesh] OR "Reproducibility of Results"[Mesh] OR “Predictability”[tiab] OR "Vertical Dimension"[Mesh] OR "Data Accuracy"[Mesh] OR "accuracy"[tiab] OR "accurate "[tiab] OR "precision"[tiab] OR "Long Term Adverse Effects"[Mesh] OR long term adverse effect*[tiab] OR "Bone Remodeling"[Mesh] OR bone remodel*[tiab] OR “Patient Satisfaction”[Mesh] OR “Patient Preference”[Mesh] OR “Relapse”[tiab] OR "stability"[tiab] OR "skeletal stability"[tiab] OR "dental stability"[tiab] OR "reliability"[tiab] OR "surgical outcome"[tiab]	

The search terms are mentioned for the following databases: Medline, Embase, Web of Science and Cochrane

### Selection of studies

Two independent reviewers (first and second author) carried out the study selection. After applying the search terms, duplicates were removed. The remaining records were screened in Covidence systematic review software (Veritas Health Innovation, Melbourne, Australia). Title and abstract screening was performed using the predefined inclusion and exclusion criteria. Any disagreements were resolved through discussion with a third author. Full-text screening followed, and reference lists of eligible articles were searched for additional relevant studies.

Data extraction was conducted independently by two authors. Extracted information included: author(s) and country of origin; year of publication; study design; number, age, and gender of patients; surgical details; methods of registration and assessment of planning accuracy and stability; reliability analysis; outcome dimensions; and results. Outcomes were qualitatively assessed and synthesized.

### Methodological quality assessment

Two observers independently assessed the risk of bias (RoB). Non-randomized studies were evaluated with the ROBINS-I tool [25], which classifies bias into seven categories, each rated as low, moderate, serious, critical, or no information. An overall risk of bias was then determined for each study (Table 2). Randomized studies were evaluated with the revised Cochrane risk of bias assessment tool [45], which covers five domains, with bias rated as low, some concerns, or high. Overall RoB was determined based on the domain evaluations (Table 3). Disagreements between observers were resolved through discussion with a third author. The overall quality of evidence was appraised using the GRADE approach.

**Table 2:** Assessment of the risk of bias for non-randomized studies (ROBINS-I tool)

		**Bias Due to Confounding**	**Bias in Selection of Participants**	**Bias in Classification of Interventions**	**Bias due to deviations from Intended Interventions**	**Bias due to Missing Data**	**Bias in Measurement of Outcomes**	**Bias in Selection of Reported Results**	**Overall bias**
1	*Centenero *et al*. (2012) * [26]	Moderate	Low	Low	Low	Low	Low	Moderate	Moderate
2	*Yao *et al*. (2015) * [27]	Critical	low	Low	Low	Low	Low	low	Critical
3	*Stokbro *et al*. (2016)* [28]	Moderate	Low	Low	Low	Low	Low	Low	Moderate
4	*Kim *et al*. (2018) *[29]	Critical	Low	Low	Low	Low	Low	Low	Critical
5	*Stokbro *et al*. (2017)* [30]	Moderate	Critical	Low	Low	Low	Low	Low	Critical
6	*Lian *et al*. (2018)* [31]	Critical	Low	Low	Low	Low	Low	Low	Critical
7	*Tankersley *et al*. (2019) * [32]	Moderate	Low	Low	Low	Low	Low	Low	Moderate
8	*Kwon *et al*. (2020) * [33]	Moderate	Low	Low	Low	Low	Low	Low	Moderate
9	*Pietzka *et al. *2020* [34]	Low	Moderate	Low	Low	Low	Low	Low	Moderate
10	*Da Costa Senior *et al*. (2021) * [35]	Moderate	Low	Low	Low	Low	Low	Low	Moderate
11	*Holte *et al*. 2021 * [36]	Moderate	Low	Low	Low	Low	Low	Low	Moderate
12	*Tanaka *et al. *2021* [37]	Moderate	Moderate	Low	Low	Low	Low	Low	Moderate
13	*Ying *et al. *2021 * [38]	Low	Moderate	Low	Low	Low	Low	Low	Moderate
14	*Chu *et al. *2022* [39]	Moderate	Moderate	Low	Low	Low	Low	Low	Moderate
15	*Holte *et al*. 2022* [40]	Moderate	Low	Low	Low	Low	Low	Low	Moderate
16	*Rios *et al. *2022* [41]	Moderate	Low	Low	Low	Low	Moderate	Low	Moderate
17	*Kuehle *et al. *2023* [42]	Moderate	Low	Low	Low	Low	Moderate	Low	Moderate
18	*Han *et al. *2024* [43]	Moderate	Moderate	Low	Low	Low	Moderate	Low	Moderate
19	*Kato *et al*. 2024* [44]	Moderate	Moderate	Low	Low	Low	Low	Low	Moderate

**Table 3:** revised Risk of Bias (RoB-2) tool for randomized controlled trials

		**Bias arising from the randomization process**	**Bias due to deviations from intended interventions**	**Bias due to missing outcome data**	**Bias in measurement of outcomes**	**Bias in selection of the reported result**	**Overall RoB judgement**
1	*De Riu *et al*. (2014) * [16]	Some	Some	low	low	low	Some
2	*Bengtsson *et al*. (2017) * [8]	Low	Low	low	Low	Low	Low
3	*Bengtsson *et al*. (2018) * [46]	Some	Low	low	Low	Low	Some

### Protocol

This systematic review was conducted in accordance with the PRISMA (Preferred Reporting Items for Systematic Reviews and Meta-Analyses) guidelines. The review protocol was registered in PROSPERO (International Prospective Register of Systematic Reviews) under the identification number CRD42018111854.

### Data extraction

Planning accuracy was defined as the discrepancy between the virtually planned position of the maxilla and their achieved position on immediate postoperative imaging, reflecting the precision of intraoperative transfer of the surgical plan. Postoperative stability was defined as the positional change of the maxilla between the immediate postoperative position and subsequent follow-up imaging, representing skeletal relapse or adaptation over time. In both cases, outcomes can be quantified without superimposition (registration-free) or using three-dimensional superimposition techniques such as landmark-based, surface-based or voxel-based registration. Reporting of the reliability of the applied 3D methodology was assessed and outcome in translational, rotational and transverse dimensions were extracted.

## Results

### Study selection

**Fig. 1:**
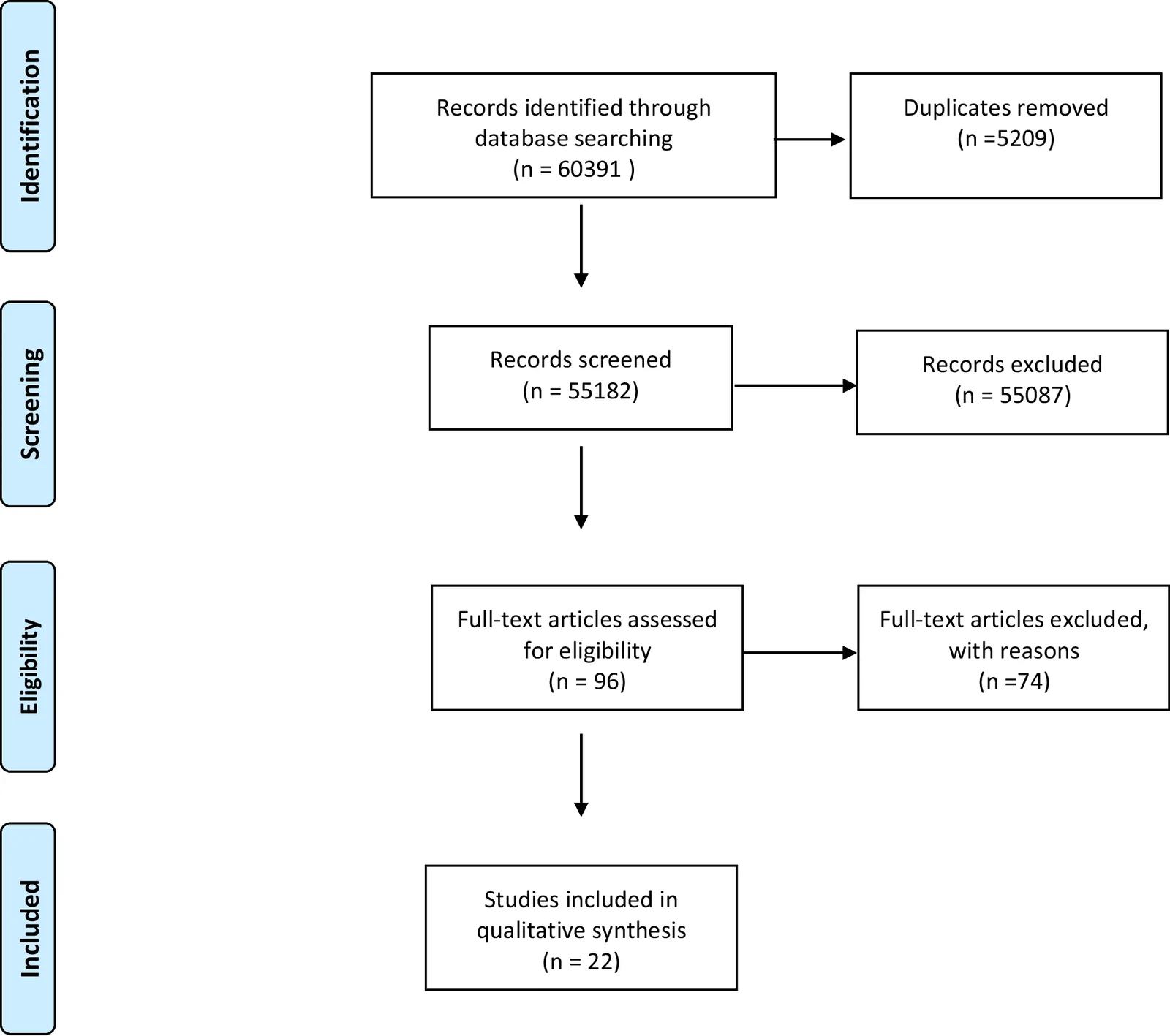
PRISMA flow chart

Database screening yielded 60,391 articles. After removal of 5,209 duplicates, 55,182 articles were screened by title and abstract. Initial disagreement on 133 studies was resolved through discussion. Ninety-six articles were selected for full-text review. One study was excluded for reporting segmental maxillary osteotomy without Le Fort I exécution [47]. Parizotto et al. [48] were excluded because stability was assessed only on 3D-generated virtual casts, and Holte et al. [26] because they analyzed differences between registration methods without reporting outcomes. Ultimately, 22 studies were included [9, 26–44, 46, 49, 50]. Reference screening revealed no additional articles. Publications spanned 2014–2024, with 19 non-randomized cohort studies [26–38, 40–44, 46, 49, 50] and three randomized controlled trials [9, 28] (Fig. 1).

### Patient and surgical characteristics

Across all studies, 496 patients underwent sLFI (Table 4). Two studies [28, 32] did not report patient numbers, and two studies [30, 46] included the same 12 patients. Seven authors were contacted for additional details such as sLFI subgroup numbers, outcome stratification, or assessment methodology; only two responded and provided extra data [9, 33].

**Table 4:** Patient and surgical characteristics

Authors	Year	Design	n/N	Age (yrs, M:F)	Skeletal class	Segment type	Planning-to-Surgery Transfer Methods	Aim
Centenero et al	2012	Prospective	5/16	NS	Class II–III	NS	CAD/CAM surgical splints	To assess precision of 3D planning
De Riu et al	2014	Prospective	NS/20	NS	NS	NS	Conventional or CAD/CAM surgical splint	To compare 2D vs 3D accuracy for correction of facial asymmetry
Yao et al	2015	Prospective	9/13	28.4 (5:4)	NS	2- or 3-piece	Model surgery splint	To compare transverse expansion in SARPE vs sLFI
Stokbro et al	2016	Retrospective	13/30	NS	NS	3-piece (most)	CAD/CAM surgical splint	To assess precision of 3D planning
Stokbro et al	2017	Retrospective	42/42	NS (16:26)	NS	3-piece	CAD/CAM surgical splint with palatal extension	To evaluate influence of splint design on transverse expansion in sLFI
Bengtsson et al	2017	Prospective	7/57	NS	Class III	NS	Not mentioned	To compare accuracy of 2D vs 3D virtual planning
Kim et al	2017	Retrospective	36/36	24.5 (10:26)	Class III	NS	Not mentioned	To evaluate transversal stability after sLFI
Lian et al	2018	Retrospective	14/37	NS	Class III	NS	CAD/CAM surgical splint	To assess stability in surgery-first sLFI
Bengtsson et al	2018	Prospective	NS/30	NS	Class III	NS	Not mentioned	To compare accuracy of 2D vs 3D virtual planning
Tankersley et al	2019	Retrospective	7/15	19	NS	3-piece (7 pts)	CAD/CAM surgical splint	To evaluate accuracy of virtual planning
Kwon et al	2020	Retrospective	63/96	20.1 (27:36)	NS	2- and 3-piece	CAD/CAM surgical splint	To assess accuracy of 3D segmental surgical planning
Pietzka et al	2020	Retrospective	9/35	25.4 (19:16)	NS	2- and 3-piece	CAD/CAM surgical splint in combination with temporary mandibular fixation to the midface	To assess accuracy of 3D-planned positioning
Rios et al	2022	Retrospective	22/22	27.4 (7:15)	Class II–III	2- and 3-piece	PSI guides and PSI plates (splintless)	To evaluate accuracy of sLFI with patient-specific plates
Holte et al	2021	Retrospective	10/10	24.4 (6:4)	Class II–III	3-piece	CAD/CAM surgical intermediate splint	To validate semi-automated 3D stability assessment in sLFI
Tanaka et al	2021	Not specified	6/6	20.8 (0:6)	NS	NS	Intra-operative navigation (Brainlab) and CAD/Cam surgical splint	To assess precision of repositioning using intra-operative navigation in sLFI
Ying et al	2021	Retrospective	20/20	25.0 (4:16)	Class I–III	NS	CAD/CAM surgical splint	To evaluate planning accuracy of sLFI
Da Costa Senior et al	2021	Retrospective	20/20	29 (7:13)	NS	2- and 3-piece	CAD/CAM surgical splint and pre-bended palatal arch bar	To validate planning accuracy and stability tool for sLFI
Chu et al	2022	Retrospective	102/129	24.5 (35:94)	Class I–III	2- to 4-piece	CAD/CAM surgical splint	To assess accuracy of sLFI
Kuehle et al	2023	Retrospective	50/50	27.8 (22:28)	Class II–III (1 Class I)	2- and 3-piece	PSI guides and PSI plates (splintless)	To quantify accuracy of PSI-based vs conventional osteosynthesis in sLFI
Holte et al	2022	Retrospective	10/10	24.4 (6:4)	Class II–III	Combined 3-piece Le Fort I + BSSO + genioplasty	CAD/CAM surgical intermediate splint	To validate a semi-automated approach for 3D assessment of stability of sLFI
Kato et al	2024	Retrospective	39/163	NS (13:26)	Class II	3-piece	Palatal splint	To compare skeletal stability of 3-piece vs 1-piece osteotomy
Han et al	2024	Retrospective	12/57	NS	NS	3-piece	Not mentioned	To validate angular accuracy of VBR for sLFI and genioplasty

Authors, prospective or retrospective study design is described. The number of segmented Le Fort I osteotomy patients (n) and total number of patients (N) included in the studies are shown. Mean age, skeletal class, number of male (M) and female (F) segmented Le Fort I (sLFI) patients and surgical specifications are depicted. The main aim of the individual studies is described. NS = not specified, SARPE = surgically-assisted rapid palatal expansion

### Methodology

**Fig. 2:**
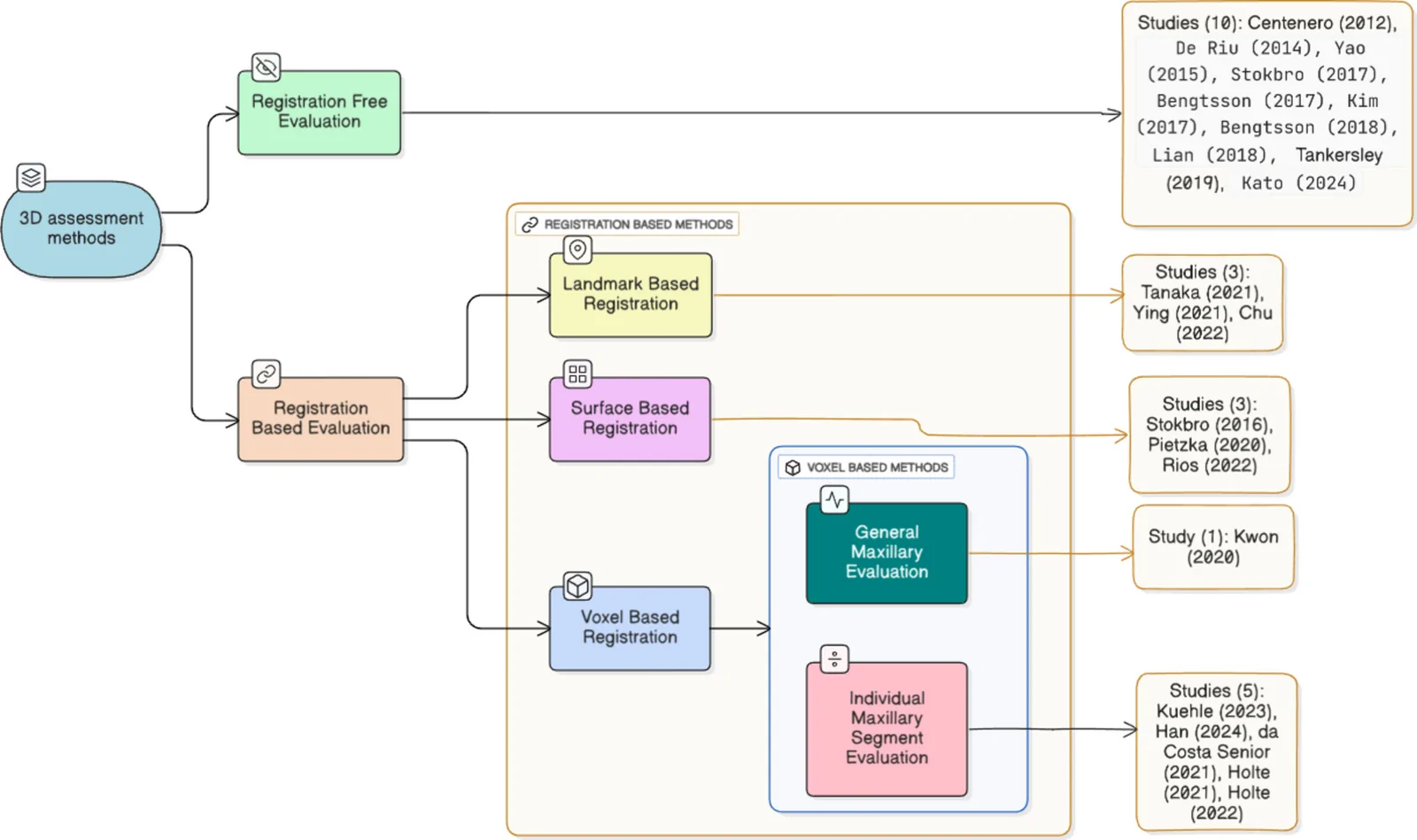
3D assessment methods. The applied 3D assessment methods were subdivided into registration-free and registration-based analysis. Registration-based assessment was further categorized as landmark-based, surface-based and voxel-based (general or individual maxillary segment) analysis

Three-dimensional assessment methods were classified as registration-free or registration-based, the latter subdivided into landmark-, surface-, and voxel-based registration. Voxel-based methods were further categorized by whether the entire maxilla or each segment was analyzed (Fig. 2). Outcomes were measured using skeletal and dental landmarks, and subdivided into translational movement (antero-posterior, medio-lateral, supero-inferior), rotational analysis (pitch, roll, yaw), and transverse width changes.

Studies assessing 3D planning against immediate postoperative imaging were classified as planning accuracy studies, while those comparing two postoperative time points were categorized as stability studies (Table 6).

### Planning accuracy

Fifteen studies reported planning accuracy [9, 28, 32, 33, 35–43, 46, 50]. Four used voxel-based registration and three surface-based methods (Table 5). Six demonstrated high reproducibility of their accuracy assessments [27, 38, 41, 43, 46].

**Table 5:** Methodology of planning, surgical accuracy and stability assessment

**Study**	**Accuracy—Stability**	**Method: registration-free or registration-based (landmark-surface-voxel))**	**Method: variable points**	**Reliability method**	**Reliability outcome**	**Outcome dimensions (translational****, rotational****, transversal****)**
ACCURACY						
Centenero et al. 2012 [26]	Accuracy	Registration: land-mark based	Dental	✖	✖	no Pitch no
De Riu et al. 2014 [16]	Accuracy	Registration-free: 3D cephalometry	Dental and skeletal	✖	✖	Left/right : no : no
Stokbro et al. 2016 [28]	Accuracy	Surface-based registration	Dental	✖	✖	Anterior/Posterior, Superior/Inferior Pitch yes
Stokbro et al. 2017 [30]	Accuracy	Registration-free	Dental	✔	✔	no no yes
Tankersley et al. 2019 [32]	Accuracy	Voxel-based registration	Dental	✖	✖	Anterior/Posterior, Medial/Lateral, Superior/Inferior no no
Kwon et al. 2020 [33]	Accuracy	Voxel based registration	Dental	✔	✔	Anterior/posterior, right/left, inferior/superior no yes
Pietzka et al. 2020 [34]	Accuracy	Surface based registration	Dental	✖	✖	Anterior/posterior, right/left, inferior/superior no no
Holte et al. 2021 [36]	Accuracy	Voxel-based registration – individual maxillary segments	Dental and skeletal	✔	✔	Anterior/posterior, right/left, inferior/superior pitch, roll, yaw no
Tanaka et al. 2021 [37]	Accuracy	Landmark based registration	Dental and skeletal	✔	✖	Anterior/posterior, right/left, inferior/superior no no
Ying et al. 2021 [38]	Accuracy	Landmark based registration	Dental and skeletal	✖	✖	Anterior/posterior, right/left, inferior/superior no no
Chu et al. 2022 [39]	Accuracy	Landmark based registration	Dental and skeletal	✔	✔	anterior–posterior, right-left and superior-inferior pitch, roll, yaw no
Rios et al. 2022 [41]	Accuracy	Surface based registration	Dental and skeletal	✖	✖	Anterior/posterior, right/left, inferior/superior no no
Kuehle et al. 2023 [42]	Accuracy	Voxel-based registration – individual maxillary segments	Skeletal	✔	✔	Anterior/posterior Left/right Superior/inferior pitch, roll, yaw yes
Han et al. 2024 [43]	Accuracy	Voxel-based registration – individual maxillary segments	Skeletal	✖	✖	no pitch, roll, yaw no
Bengtsson et al. 2017 [8]	Accuracy/Stability Follow-up: 12 months	Registration-free: 3D cephalometry	Dental and skeletal	✔	✔	Anterior/Posterior, Superior/Inferior Pitch no
Bengtsson et al. 2018 [46]	Accuracy/Stability Follow-up: 12 months	Registration-free: 3D cephalometry	Dental and skeletal	✔	✔	Anterior/Posterior, Superior/Inferior Pitch no
da Costa Senior et al. 2021 [35]	Accuracy	Voxel based registration – individual maxillary segments	Skeletal	✔	✔	Anterior/posterior Left/right Superior/inferior pitch, roll, yaw no
STABILITY						
Yao et al. 2015 [27]	Stability Follow-up: 6 months	Registration-free	Dental and skeletal	✖	✖	no no yes
Kim et al. 2018 [29]	Stability Follow-up: 12 months	Registration-free	Dental and skeletal	✔	✔	no no yes
Lian et al. 2018 [31]	Stability Follow-up: until bracket removal	Registration-free: 3D cephalometry	Sketetal	✖	✖	(B-point): anteroposterior superior/inferior no no
da Costa Senior et al. 2021 [35]	Stability Follow-up: 6 months	Voxel based registration – individual maxillary segments	Skeletal	✔	✔	Anterior/posterior Left/right Superior/inferior pitch, roll, yaw no
Holte et al. 2022 [40]	Stability Follow-up: 24 months	Voxel-based registration – individual maxillary segments	Dental and skeletal	✔	✔	anterior–posterior, right-left and superior-inferior pitch, roll, yaw no
Kato et al. 2024 [44]	Stability Follow-up: ≥ 10 months	Registration-free: 3D cephalometry	Dental and skeletal	✔	✔	3D Euclidean distances

Assessment methods were classified as either registration-free or registration-based on the non-operated skull. Registration-based methods were further divided into landmark-, surface-, and voxel-based approaches. Outcome variables were measured at dental or skeletal reference points. The use of reliability analysis was indicated where reported. Outcome dimensions were grouped into translational, rotational, and transverse measurements. ✔ = reported; ✖ = not reported

Translational accuracy was reported in 11 studies [17, 27, 30, 33, 34, 37, 38, 41–43]. Most found advancement to be the least accurate movement (0.09—3.24 mm inaccuracy), though significance was confirmed only by Tankersley et al. [33] and Kwon et al. [36]. Rotational accuracy was analyzed in six studies [16, 16, 16, 30, 38, 40, 41, 43], with Stokbro et al. [30] reporting a significant clockwise pitch rotation (*P* = 0.006; −0,54° clockwise rotation). Four studies investigated transverse expansion [27, 30, 43, 46] (Table 6). Stokbro et al. [30] found a mean underachievement of 1.41 mm, which improved to 0.77 mm when a splint with high palatal coverage was used [30, 46]. Using a voxel-based approach, Da Costa Senior et al. [36] demonstrated that advancement and pitch were the least accurately achieved movements, with discrepancies ranging from 1.04 to 2.52 mm and 3.36° to 5.03°, respectively. Kwon et al. [27] reported 0.96 ± 0.69 mm transverse accuracy after 2- and 3-piece osteotomies. Kuehle et al. [43] found no significant translational difference in transverse accuracy with patient-specific osteosynthesis compared to conventional fixation, although inward segmental rotation was greater in the conventional group (up to 2.08°).

**Table 6:** Outcome of accuracy of planned segmental Le Fort I osteotomy and stability

Study	Method of outcome reporting	Outcome	Significance (P-value)
ACCURACY			
Centenero et al. 2012 [26]	ICC (between planning and achieved movement)	pl_Fr_Oc: 0,375 ^1^ Ns _Bs_A 0,814^1^	pl_Fr_Oc: 0.085^1^ Ns _Bs_A: 0.933^1^
De Riu et al. 2014 [16]	Rate of alignment (percentage)	Upper inter incisal point-facial midline: 88.10 Maxillary sagittal plane-facial midsagittal plane: 71.61	Upper inter incisal point-facial midline: 0.41 Maxillary sagittal plane-facial midsagittal plane: 0.39^1^
Stokbro et al. 2016 [28]	MLD/MAD	*Linear differences*: Anterior/Posterior: −0.87 mm Medial/Lateral: −0.15 mm Superior/Inferior: −0.10 *Rotational differences*: Pitch: −0,54° (clockwise) Roll: −0.03° Yaw: 0.17° *Transversal*: −1.41 mm	*P-values* Anterior/Posterior: 0.290 Medial/Lateral: 0.567 Superior/Inferior: 0.779 *Rotational differences*: Pitch: 0.006 Roll: 0.820 Yaw: 0.072 *Transversal*: 0.002
Stokbro et al. 2017 [30]	MLD	Transversal: −0,77 mm (SD 0.83)	*P* < 0.01
Tankersley et al. 2019 [32]	Root mean square deviation	*Maxillary centroid* Anteroposterior: 2.4 mm Transverse: 1.1 mm Vertical: 0.7 mm *Maxillary incisor* Anteroposterior: 2,6 mm Transverse: 1.3 mm Vertical:1.2 mm	*Maxillary centroid* Anteroposterior: < 0.05 Transverse: > 0.05 Vertical: > 0.05 *Maxillary incisor* Anteroposterior: < 0.05 Transverse: > 0.05 Vertical: > 0.05
Kwon et al. 2020 [33]	(Absolute) MLD	*Absolute mean* Left–right: 0.96 mm Superior-Inferior 1.23 mm Anteroposterior: 1.16 mm Intercanine width: 0.95 mm Intermolar width: 1.22 mm	Left–right: < 0.001 Superior-Inferior: < 0.001 Anteroposterior: < 0.001 Intercanine width: 0.002 Intermolar width: < 0.001
Pietzka et al. 2020 [34]	Median, maximum, minimum, range, and interquartile range	absolute median difference: 1.13 mm median difference: −0.61 mm (over 3 axes and 5 landmarks)	NS
Holte et al. 2021 [36]	MD and MAD	Absolute mean: Anterior/Posterior: 1.17—1.50 mm Medial/Lateral: 0.91—1.23 mm Superior/Inferior: 0.55—0.87 mm Pitch: 1.93—5.22° Roll: 1.53—5.84° Yaw: 2.30—2.42°	NS
Tanaka et al. 2021 [37]	Absolute median difference	Anterior/Posterior: 0.09—3.24 mm Medial/Lateral: 0.29—1.51 mm Superior/Inferior: 0.01—0.45 mm	*P* > 0.05 (not significant)
Ying et al. 2021 [38]	MD	Anterior/Posterior: −0.88—0.78 mm Medial/Lateral: −0.42—0.28 mm Superior/Inferior: −0.65—0.27 mm	*P* > 0.05 (not significant)
Chu et al. 2022 [39]	linear differences (mm) and angular differences (°)	MD: Anterior/Posterior: 0.92—2.69 mm Medial/Lateral: 0.76—1.67 mm Superior/Inferior: 0.74—1.53 mm Pitch: 1.73—2.14° Roll: 2.11—2.86° Yaw: 1.87–3.19°	Posterior vertical accuracy (*P* < 0.05)
Rios et al. 2022 [41]	MAD	Anterior/Posterior: 0.74 mm Medial/Lateral: 0.59 mm Superior/Inferior: 0.56 mm	NS
Kuehle et al. 2023 [42]	MD	MD: Anterior/Posterior: −1.36—−0.49 mm Medial/Lateral: −0.63—0.17 mm Superior/Inferior: −3.22—−1.20 mm Pitch: 0.12—3.57° Roll: −0.05—2.08° Yaw: −2.12—1.30°	NS
Han et al. 2024 [43]	Mean absolute angular error	Anterior maxilla (MAD): Pitch: 0.20–0.38° Roll: 0.28—0.40° Yaw: 0.34—0.54°	all *P* ≤ 0.01 (*P* < 0.05 was considered significant)
Bengtsson et al. 2017 [8]	(Absolute) MLD/MAD	MAD SNA-S2N2A2: 1.36° 11/NSL-112/NSL2: 0.23° A-A2: 1.86 mm	Absolute mean difference: SNA-S2N2A2: < 0.001 11/NSL-112/NSL2: < 0.001 A-A2: < 0.001
Bengtsson et al. 2018 [46]	MLD/MAD	Mean difference: SNA-S2N2A2: 1.03° 11/NSL-112/NSL2: −0.14° A-A2: 1.77 mm	Nonabsolute mean difference: SNA-S2N2A2: 0.0019 11/NSL-112/NSL2: 0.033 A-A2: < 0.001
Da Costa Senior et al. (2021) [35]	(Absolute) MLD/MAD	Absolute mean: Anterior/Posterior: 1.04—2.52 mm Medial/Lateral: 0.70—2.14 mm Superior/Inferior: 0.84–1.56 mm Pitch: 3.36—5.03° Roll: 2.19—5.29° Yaw: 2.12—2.99°	NS
STABILITY			
Yao et al. 2015 [27]	Absolute MLD; immediate postoperative – 6 months postoperative	Posterior skeletal: −0,86 mm Anterior skeletal: −0,55 mm Intermolar width: −0,77 mm Intercanine width: −0,39 mm (SD 1.01)	Posterior skeletal: < 0.0005 Anterior skeletal: < 0.05 Intermolar width: < 0.0005 Intercanine width: < 0.005
Kim et al. 2018 [29]	MLD; immediate postoperative – end of treatment (12.78 months, SD 4.26)	Transversal dental width: −1,41 mm Transversal skeletal width: −0,67 mm	Transversal dental width: < 0.001 Transversal skeletal width: < 0.001
Lian et al. 2018 [31]	MLD; immediate postoperative – end of treatment	B-point: Anteroposterior: 1.80 mm Superior/inferior: 1.34 mm	NS
Da Costa Senior et al. (2021) [35]	(Absolute) MLD/MAD	Absolute mean: Anterior/Posterior: 0.40–1.30 mm Medial/Lateral: 0.31–1.32 mm Superior/Inferior: 0.37–1.41 mm Pitch: 1.29—3.57° Roll: 1.30—1.92° Yaw: 0.73—1.16°	NS
Holte et al. 2022 [40]	MD and MAD	Absolute mean: Anterior/Posterior: 0.58—1.52 mm Medial/Lateral: 0.51—1.39 mm Superior/Inferior: 1.02—1.16 mm Pitch: 1.30—3.37° Roll: 1.87—3.15° Yaw: 1.00—1.22°	NS
Kato et al. 2024 [44]	MD with follow-up of > 10 months	Anterior skeletal width: −0.39 mm Posterior skeletal width: 0.05 mm	*P* > 0.05

Methodology, outcome measurements and significance are described. NSL = sella-nasion line; MD = mean difference; MAD = mean absolute difference; MLD: mean linear difference. pl_Fr_Oc = angle formed between the Frankfurt Plane and the Occlusal Plane. Angle Ns_Bs_A = Bs: most anterior point of the foramen magnum. A: most concave point and medial point at the maxilla bone level

### Surgical stability

Six studies evaluated postoperative stability [26, 29, 31, 35, 44, 49]. Three validated their methods with intra-observer testing [26, 44, 51]. Two studies assessed transverse stability using 3D skeletal and dental landmarks [29, 31]. Kim and Cha [31] observed an intermolar relapse of 1.41 mm and skeletal narrowing of 0.67 mm after ~ 13 months, with splint retention for one month postoperatively. Yao et al. [29] reported significant skeletal transverse relapse both anteriorly (*P* < 0.05) and posteriorly (*P* < 0.0005), with dental relapse of −0.77 mm at the molars and −0.39 mm at the canines. Da Costa Senior et al. [36] analyzed stability of individual maxillary segments up to 6 months postoperatively and found relapse in the pitch dimension between 1.29—3.57°. Holte et al. [44] reported greatest relapse in anteroposterior translation (0.58—1.52 mm) and pitch rotation (1.30—3.37°), but not in transverse width. Bengtsson et al. [9, 32] compared one-year postoperative results with preoperative planning using 3D cephalometry. Their method proved reliable [32], but results showed a significant posterior shift of the A-point relative to planning. As these studies did not differentiate between segmental and one-piece Le Fort I or surgical inaccuracy or postoperative instability, findings must be interpreted cautiously.

Due to heterogeneity in assessment methods and surgical techniques, a meta-analysis was not feasible. The PRISMA checklist was applied (Fig. 1).

### Risk of bias and quality of evidence

Using ROBINS-I, four studies [29, 31, 46, 49] were deemed at critical risk of bias and fifteen at moderate risk [16, 26, 27, 30, 33, 34, 36–38, 40–44] had a moderate risk of bias (Table 2). ROB-2 identified some concerns for two randomized studies [28, 32] and low risk in one [9] (Table 3). GRADE showed a moderate quality of evidence for the randomized studies and low to very low quality of evidence for the cohort studies.

## Discussion

The stability of sLFI has long been questioned in the literature. Proffit et al. [12] ranked transverse stability as the least reliable in their hierarchy of surgical stability, a reputation largely based on two-dimensional (2D) anteroposterior cephalograms and dental cast analyses. Consequently, these earlier analyses were unable to fully capture the complex, multi-dimensional behavior of segmented maxillary movements. With the advent of 3D imaging, planning, and follow-up, a more comprehensive evaluation of orthognathic surgery has become possible. Over the past decade, there has been a marked increase in studies applying 3D assessment to Le Fort I osteotomies. However, until now, no systematic review has specifically focused on 3D outcome assessment in sLFI. By synthesizing this emerging body of literature, the present review provides a more detailed and mechanistic understanding of surgical accuracy and postoperative stability, thereby extending the findings of earlier reviews.

The aim of this systematic review was to evaluate the 3D assessment of sLFI, focusing on both surgical planning accuracy and postoperative stability. A secondary objective was to examine the diversity of current 3D assessment methods applied. After screening, 22 articles were included, of which five were prospective studies and three were randomized controlled trials. Fifteen studies reported on surgical accuracy and six on postoperative stability with two studies directly comparing surgical planning with long-term follow-up.

### Accuracy

Fourteen studies assessed whether planned movements were achieved. For the transverse dimension, multiple studies found the transverse dimension difficult to achieve. Stokbro et al. [30, 46] proposed a planned overexpansion strategy and modified the splint with high palatal coverage, which significantly reduced this underachievement. Kuehle et al. [43] reported a pronounced endorotation of the lateral maxillary segments after evaluating the individual maxillary segments. This endorotation could influence the achieved transverse width and might explain why a high palatal coverage splint can improve this involuntary rotation and subsequent transverse underachievement. It seems that adequate control of the roll of the lateral segments could play an important role in optimizing surgical outcome. This finding highlights the added insights gained by 3D evaluation of the individual segments.

In the sagittal dimension, advancement was consistently the least accurately achieved translational movement, in line with findings from non-segmented Le Fort I osteotomy [52, 53]. This suggests that segmentation itself does not inherently compromise translational accuracy, but rather that advancement remains a general surgical challenge.

Rotational accuracy was reported in five studies. Pitch consistently emerged as the most difficult rotation to control. Importantly, accuracy values tend to appear larger when individual maxillary segments are analyzed [35, 50]. This finding is not unexpected, as limited inaccuracies in individual maxillary segments will lead to larger rotational discrepancies as the analyzed volume is smaller. Readers should keep this in mind, when interpreting rotational outcome values. Kuehle et al. [43] demonstrated that PSI significantly improved pitch control compared with conventional fixation.

### Stability

Postoperative stability was assessed in six studies. Significant transverse skeletal and dental relapse was observerd, with dental relapse being more pronounced [8, 54]. Relapse was attributed to soft tissue rebound, buccal musculature pressure, and palatal tissue forces. Yao et al. [29] also compared sLFI with surgically assisted rapid palatal expansion (SARPE), showing relatively greater skeletal versus dental expansion in sLFI, though SARPE is often considered more stable—a conclusion largely based on 2D analyses [53, 54]. Holte et al. [44] evaluated stability of individual segments and found anteroposterior relapse most likely. The vertical stability of the anterior segment showed very limited intrusion relapse—statistically detectable but not clinically significant in open bite corrections—while the lateral segments tended to flare outward, consistent with the accuracy findings of Kuehle et al. [43]. This last finding could explain the larger dental than skeletal relapse and helps understanding the biomechanical mechanism underlying clinical findings.

Several studies tested alternatives to conventional protocols. Stokbro et al. [32] confirmed that high-coverage palatal splints improved transverse accuracy. Kuehle et al. [43] compared PSI with conventional osteosynthesis and found that the PSI group showed significantly less deviation from the planned position in the anteroposterior, craniocaudal and rotational dimensions. PSI also improved pitch control and reduced inward rotation of the lateral segments. Tanaka et al. [38] piloted intraoperative navigation in six patients, achieving encouraging accuracy results.

### 3D assessment protocols

Large variability exists regarding 3D assessment strategies, which led to the proposed assessment protocol by Gaber et al. [23] to reduce the possibility of error. It was recommended to perform voxel-based registration, (semi-)automated outcome evaluation and inter-and intra-observer reliability. In this review, only five included studies adhered to these recommendations [35, 36, 43, 44, 50]. The more recent included publications increasingly adopted voxel-based registration and even evaluated the positional outcome of individual maxillary segments. In addition to the recommendations by Gaber, we recommend separate reporting of surgical accuracy (where planning is compared with immediate postoperative scans) and stability where postoperative scans are compared. This distinguishes surgical error from postoperative remodelling and relapse. Furthermore, we recommend reporting of the individual maxillary segments as this will further our understanding of this complex procedure and ultimately help improve outcome.

The findings of the present review give a clear added insight in relation to the previous review by Haas et al. [7]. Current findings provide a higher-resolution, three-dimensional interpretation of segmental behavior. Whereas Haas et al. relied primarily on two-dimensional measurements and aggregated outcomes, the present review highlights that postoperative changes are not solely translational but are strongly influenced by rotational discrepancies, particularly in pitch and roll. Furthermore, by distinguishing between surgical accuracy and postoperative stability, the present review gives a broader overview of skeletal outcome in contemporary orthognathic surgery. Importantly, segment-specific analyses demonstrate that lateral and anterior maxillary segments exhibit different patterns of behaviour, both intraoperatively and during follow-up. These findings provide a more mechanistic understanding and refine the clinical interpretation of outcome after segmented Le Fort I osteotomy.

### Evidence quality

Risk of bias assessment with ROBINS-1 and ROB-2 revealed that most studies were of moderate to critical risk, with GRADE ratings ranging from moderate (randomized studies) to low/very low (cohort studies). Nearly all studies were limited by confounding and reporting bias, small sample sizes, or retrospective design. In two, the exact number of sLFI patients was not specified, preventing clear subgroup analysis. Heterogeneity in 3D assessment methods further precluded meta-analysis.

## Conclusion

This systematic review demonstrates that sLFI can achieve a high degree of accuracy and acceptable postoperative stability, yet specific challenges remain. Surgical advancement and pitch control, particularly the tendency toward inward rotation of the lateral segments, continue to represent weak points in surgical precision. Evidence indicates that patient-specific implants and intraoperative navigation are promising adjuncts for improving accuracy, while postoperative relapse occurs more prominently at the dental than at the skeletal level, even with splinting. A critical finding of this review is the considerable methodological variability across studies, particularly in 3D assessment strategies, which hinders direct comparison and prevents meta-analysis. This underscores the urgent need for standardized, validated 3D protocols to reliably evaluate surgical accuracy and stability. The added value of this review lies in its consolidation of the fragmented body of evidence, highlighting consistent strengths and weaknesses of sLFI while mapping the current state of 3D outcome assessment. By emphasizing where inaccuracies occur and identifying technological advances that mitigate them, this work provides a roadmap for future refinement of surgical techniques and research methodology. Future progress in sLFI will depend on prospective, large-scale clinical trials, conducted under unified and validated 3D evaluation protocols. Only then can the field move from isolated insights to robust evidence, ultimately improving surgical reliability and patient outcomes.

## Data Availability

No datasets were generated or analysed during the current study.
